# Modeling the Drying Process of Porous Catalysts: Impact of the Pore Size Distribution

**DOI:** 10.1021/acs.iecr.3c03057

**Published:** 2023-11-14

**Authors:** David R. Rieder, Elias A. J. F. Peters, Johannes A. M. Kuipers

**Affiliations:** Multiphase Reactors Group, Department of Chemical Engineering and Chemistry, Eindhoven University of Technology, MB Eindhoven 5600, The Netherlands

## Abstract

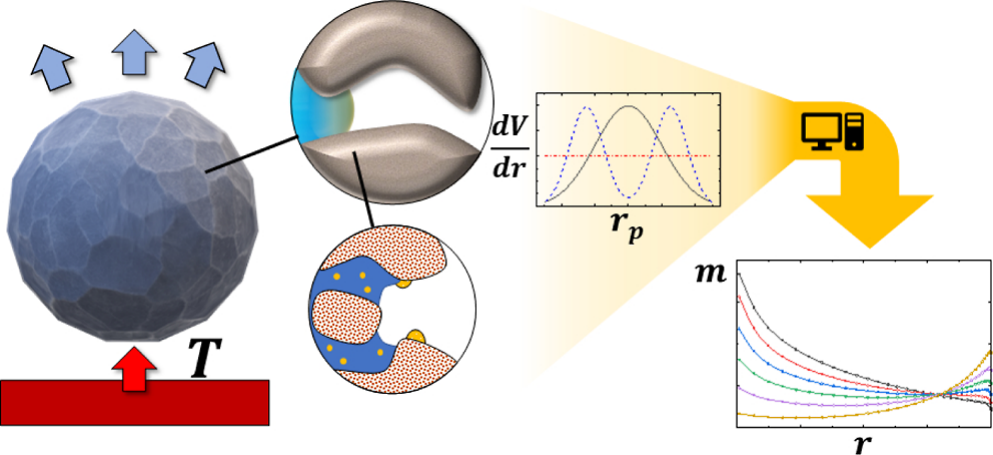

The distribution of catalytically active species in heterogeneous porous catalysts strongly influences their performance and durability in industrial reactors. A drying model for investigating this redistribution was developed and implemented using the finite volume method. This model embeds an analytical approach regarding the permeability and capillary pressure from arbitrary pore size distributions. Subsequently, a set of varying pore size distributions are investigated, and their impact on the species redistribution during drying is quantified. It was found that small amounts of large pores speed up the drying process and reduce internal pressure build up significantly while having a negligible impact on the final distribution of the catalytically active species. By further increasing the amount of large pores, the accumulation of species at the drying surface is facilitated.

## Introduction

Modern production chains rely heavily on catalysts due to their ability to accelerate chemical reactions and tune the selectivity toward the desired end product. As a result, the target processes may benefit from smaller reactors, less aggressive or toxic educts, or simply may allow for realistic time scales, improving their economic feasibility.^1^ Among the most favorable catalytically active species, precious metals such as platinum, rhodium, or gold are often encountered. Those are typically scarce resources and often mined under questionable conditions, both from an ecological and human rights view.^2,3^ Therefore, it is of high importance to maximize the efficiency of the catalytically active species within the relevant applications and reduce the required material load to a minimum.

One widespread strategy to intensify the contact between educts and catalyst can be found in the form of supported catalysts. There, distribution of the catalyst on the internal surface leads to high exposure of reaction sites and hence to higher reaction rates per unit volume of catalyst.

Within industrial scale processes, those supported catalysts are commonly employed in the form of millimeter-sized pellets of various shapes, of which the sphere is arguably the simplest. Those pellets are then used either in a static fashion in packed bed type reactors or within more dynamic systems such as fluidized beds. The total performance of any of those reactor types is heavily dependent on a variety of parameters, i.e. pressure drop, educt quality, catalyst degradation, and inter-as well as intraparticle mass- and heat-transfer limitations.^4^ Finding an optimized solution for such a process usually requires the use of a specialized catalyst distribution within the porous pellets.

Historically, various preparation possibilities for supported catalysts have been explored, of which the incipient wetness method has enjoyed significant popularity. There, the porous pellet is impregnated with a solution loaded with a catalyst precursor, subsequently dried and finally calcinated.^1^ The actual deposition of the target catalyst is influenced by all of those steps, from an atomic level within a single pore to macroscopic distribution within the pellet. As the porous materials often target high specific surface areas, their representative pore sizes usually are several magnitudes lower than the length scale of the pellet, thus posing an intricate multiscale problem. Within this work, the redistribution of the catalytic compound on the pellet level is investigated, focusing on the internal hydrodynamics and their interaction with the catalytically active species.

Initial research concerning the deposition of catalyst inside porous materials focused on the impregnation step, assuming that fast drying reduces the redistribution to a minimum.^5,6^ Especially for support–catalyst combinations with strong adsorption characteristics, these investigations provided highly relevant insights into the associated phenomena. Until today, the impregnation step is receiving significant interest, especially with the improvements in experimental methods, e.g., magnetic resonance imaging,^7,8^ UV–vis^9^ or Raman spectroscopy.^10^

However, for supported catalysts as used in industry, the adsorption during impregnation often only plays a small part in the final postdrying distribution of the active species. Commonly, the hydrodynamics induced by capillary suction during the drying step dominates the mass-transport phenomena inside the pellet, leading to redistribution of the catalytic compound.^11^ With increasing interest in the drying step and advances in averaging theory, a variety of model approaches were developed to describe the associated phenomena. Of those, the receding front model^12^ and Whitaker’s volume averaging^13^ have been investigated extensively. For a dedicated review of popular drying models, the interested reader is referred to Vu and Tsotsas.^14^

Originally developed and verified against macroporous materials, i.e., soil or wood,^15^ the volume averaged formulation has also been successfully applied for the prediction of postdrying catalyst profiles in micro- and mesoporous materials.^11,16,17^ In all those cases, the transport is highly dependent on averaged properties of the porous media, e.g., saturation, permeability, and capillary pressure to name a few. Often, those are determined experimentally for a support, yet research into analytical derivations of those parameters from routinely measured bulk properties is quite active.^18−22^ Those models often rely on dedicated configurations of the pore size distributions and therefore allow the derivation of the relevant parameters from the porosity and the knowledge of minimum and maximum pore size. One interesting exception to this approach is given by the derivation of the averaged properties from an available pore size distribution, which offers a variety of potential insights in the impact of local pore morphology onto the drying process.^23^

However, the investigations so far carried out using this approach focused mainly on the drying of light concrete soaked with pure water and provide no insight into the transport and precipitation of aqueous species. In contrast, mass transport modeling for this purpose has been conducted, relying on empirical correlations to describe the associated support material.^11,24−27^ Thus, leaving open the question of how the pore morphology influences the intrapellet mass transport and subsequently the postdrying precipitate distribution.

As a means to distinguish the impact of the pore size distribution on the drying of porous materials, it is pertinent to formulate a drying model in which the influence of other phenomena is either easily understood or can be removed altogether. For this purpose, a dedicated model is developed to allow the study of separate transport phenomena as well as their coupling with other properties and process parameters. By gradually increasing the complexity of this model, valuable insights into the relevance of each of the model assumptions, material properties, and transport phenomena can be gained, as well as their impact on the drying process understood.

To investigate the role of the pore-level morphology in the supported catalyst preparation process, the aforementioned drying model framework^23^ has been extended with mass transfer and precipitation. By inclusion of Metzger’s permeability model,^18^ various types of pore size distributions are subjected to the drying process and the final precipitation profiles determined.

## Modeling Approach

### Governing Equations and Boundary Conditions

**Figure 1:**
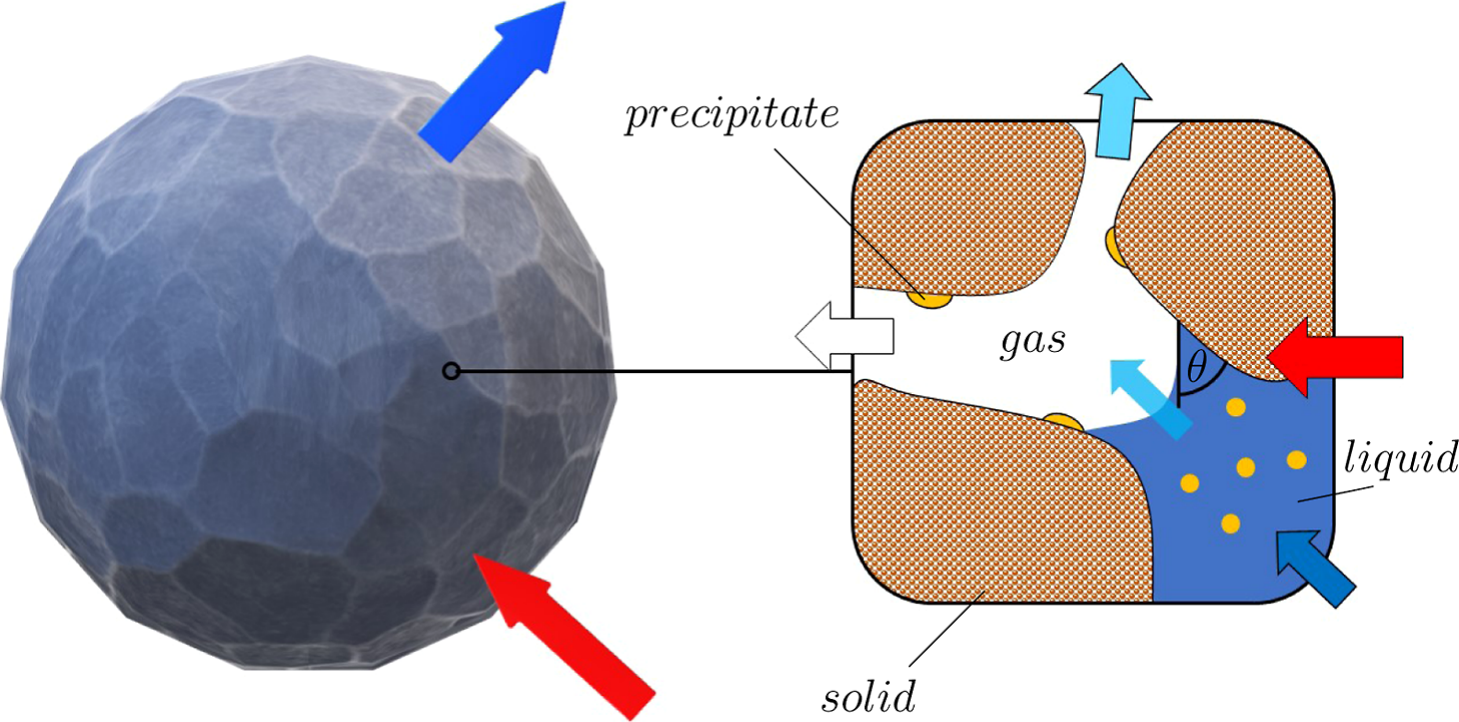
Overview of the transport phenomena coupled within the drying model at the pellet (left) and pore length (right) scale: The porous pellet is exposed to a heated gas flow, leading to a vapor flow across the external boundary. Within the pores, gas (white), gas vapor (light blue), liquid (dark blue), aqueous species (yellow), and energy transport (red) are included. Once the species precipitated within the pore, it is considered to be immobile. Within the pores, the three-phase contact line (with contact angle θ) leads to capillary suction and subsequently a flow of liquid, while evaporation leads to the local generation of vapor.

This model follows the approach presented in an earlier work, where the impact of nonlinear fluid properties was investigated.^28^ To improve the readability, the main governing equations and main assumptions are briefly reiterated here. This drying model includes the conservation of the components inert gas, volatile liquid, aqueous species, and precipitate, as well as heat transport, as shown in Figure 1. Within this work, air and water are considered as inert gases and volatile liquids in accordance with industrial practice. Nevertheless, note that the presented model is more generally applicable and can be employed for a variety of gas–liquid combinations. The main assumptions are as follows:

(i) isotropic properties of support and fluids
(ii) volume averaged equations are applicable
(iii) support is rigid and fixed in space
(iv) locally the system is in thermal equilibrium
(v) gravity is negligible
(vi) the dissolved species is nonvolatile
(vii) precipitated species is immobile

The set of governing equations for the conservation equation of water w, air a, aqueous precipitating species, precipitate p and the energy balance in eq 1 to 5 are expressed as
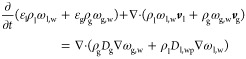
1
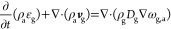
2
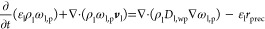
3
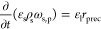
4
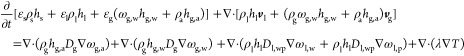
5with the volume fractions of solid, liquid, and gas phase ε_s_, ε_l_, and ε_g_, the densities of liquid ρ_l_ and gas phase ρ_g_, the mass fractions of water, air, dissolved, and precipitated species ω_l,w_, ω_g,w_, ω_g,a_, ω_l,p_, and ω_s,p_, as well as the enthalpy *h*_i_ of each component *i*. Within this model, the porous solid media, s, is considered immobile and inert. Furthermore, the effective binary diffusion coefficients of the air–vapor and water species are denoted as *D*_g_ and *D*_l,wp_. Here, the local gas–liquid–solid contact is considered to be in thermal equilibrium with the representative temperature *T*. Thermal conduction is incorporated via an effective thermal conductivity λ and convective transport is integrated via a Darcy-type of flow by the liquid and gas side velocity ***v***_l_ and ***v***_g_, explained in more detail below. For the precipitation, an effective reaction rate *r*_prec_ is incorporated, which regulates the local mass fraction to not exceed supersaturation. Within this work, the change in solid volume fraction ε_s_ as well as solid density ρ_s_ due to precipitation is not accounted for and subsequently considered constant. Note that the precipitate is considered as immobile phase and pore-scale redistribution is neglected. Precipitation is modeled in terms of the supersaturation ω_l,p_^+^, with the associated tunable rate constant *k*_prec_.
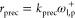
6
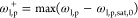
7

The drying of the pellet is conducted by exposing the outer boundary to an air stream at ambient temperature *T*_∞_ with a representative value for ambient water vapor pressure *P*_v,∞_. As the focus in this work is laid on a spherical geometry, no flux is acting in the center. Across the exposed surface, water is only allowed to flow as vapor and no flux of its liquid phase permitted, allowing to define the boundary flux as
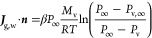
8with the external mass-transfer coefficient β, the ambient pressure *P*_∞_, the ideal gas constant *R*, and the molar weight of water *M*_v_. For the energy equation, following boundary condition is applied
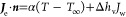
9with the external heat transfer coefficient α and the enthalpy of the vapor Δ*h*_v_. For the transport of air, ambient pressure is prescribed at the outer boundary.
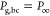
10

The sum of all mass fractions in the liquid leads to unity
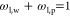
11

Furthermore, as the volume fraction of the precipitated species is considered negligible, following closure can be formulates
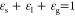
12

Thus, the liquid saturation *S*_l_ can be expressed as
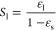
13

### Fluid Velocities and Permeability Model

For the convective transport in the pores, Darcy flow is applied to determine the liquid and gas velocities
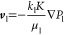
14
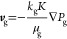
15with the partial permeabilities of the liquid and gas phase *k*_l_ and *k*_g_, the permeability of the porous medium *K* and the respective viscosity values μ_l_ and μ_g_, as well as the pressure gradients ∇*P*_l_ and ∇*P*_g_. The pressures are linked by
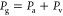
16
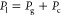
17with the capillary pressure *P*_c_. Under the assumption of an ideal gas, *P*_a_ and *P*_v_ are correlated to ω_g,a_ and ω_g,w_ via
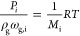
18with the molar mass *M*_i_ of species i. The capillary pressure is computed by the Young–Laplace law for a circular meniscus, assuming that the largest filled pore with pore radius *r*_p,f_ is dominating the pressure and the liquid shows a contact angle θ at the three-phase contact line with the surface tension σ
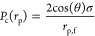
19

The total permeability *K*, as well as the partial permeabilities *k*_l_ and *k*_g_ as used in eqs 14 and 15 are determined using the approach formulated by Metzger.^18^ There, a pore space is approximated as a bundle of capillaries with varying diameters and associated volumes. This pore space is represented by the cumulative normalized pore size distribution *V*_p_, where *V*_p_ is normalized to conform to
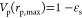
20with the pore radius *r*_p_. Comparing Hagen–Poiseuille’s law with a bundle of capillaries leads to following relationship for *K*
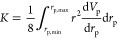
21with lower and upper limits *r*_p,min_ and *r*_p,max_, as well as the differential pore size distribution d*V*_p_/d*r*_p_. Furthermore, the partial permeabilities for the liquid and gas phases are given by
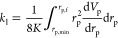
22
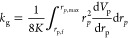
23

From eqs 22 and 23 follows
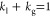
24

The largest filled pore can be found in dependence of the saturation of free liquid *S*_l,f_ of the porous media
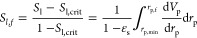
25where *S*_l,crit_ denotes the saturation content, at which a monolayer is adsorbed on the surface of the pores, convective flow stops, and only evaporation is reducing the moisture, as described in section.

### Phenomena at Low Saturation

Once the liquid saturation falls below the critical value *S*_l,crit_, drying only continues via evaporation and subsequent vapor transport. This evaporation process is modeled as an equilibrium phenomena via the adsorption isotherm used by Perré and Turner^15^
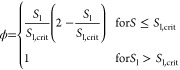
26with the saturation at the maximum amount of adsorbed water *S*_l,crit_ and the relative humidity ϕ. Note that for simplicity a dependency of the isotherm on *r*_p_ as follows from the Kelvin equation is neglected, since the deviation in ϕ is estimated to be less than 10%. The partial permeabilities at *S*_l_ < *S*_l,crit_ are given by
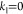
27
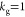
28

For the presented model, a variety of additional properties and couplings are required. For readability, those are provided in the Supporting Information and are not repeated here.

### Numerical Treatment

As a means to solve the model equations, Mathworks MATLAB was used. The governing equations are discretized by using the finite-volume approach on a 1D radially symmetric grid. For approximating the solution of the nonlinear system of partial differential equations, Newton–Raphson iterations are employed within each discrete time step, where the associated Jacobian is determined via numerical differentiation. Further details and validation of the implementation are provided in the Supporting Information.

## Results and Discussion

As a model porous support, a spherical pellet with a radius of *R* = 2 mm and a solid volume fraction of ε_s_ = 0.4 was chosen. The critical saturation was set to be *S*_l,crit_ = 0.35 and the initial mass fraction of aqueous species to ω_l,p_ = 0.1 ω_l,p,sat_. Drying itself is conducted at atmospheric pressure *P*_∞_ = 101,300 Pa, ambient water vapor pressure at *P*_v,∞_ = 0 Pa and drying temperature at *T*_∞_ = 100 °C. The pellet is considered dry once saturation at any point does not exceed 10^–6^.

For convenience, the precipitated species is expressed in terms of load on the solid porous material
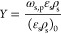
29with the initial mass of the unloaded support . More details can be found in the Supporting Information. Within this work, uniform (U), monomodal (M), and bimodal (B) distributions are investigated. For the uniform distribution, the following expression was employed
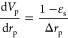
30with the width of the pore size distribution Δ*r*_p_ = *r*_p,max_ – *r*_p,min_ and the associated interval *r*_p,min_ ≤ *r*_p_ ≤ *r*_p,max_. The mono- and bimodal distributions are implemented as Gaussian standard distributions
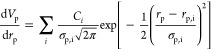
31with the number of modes i, their respective fraction volume *V*_p,i_, mean pore diameter *r*_p,i_, and standard deviation σ_p,i_.

### Distribution Width at Small Scale

As a first step, the impact of the width of the pore size distribution Δ*r*_p_ was investigated by adapting *r*_p,min_ and *r*_p,max_ of the uniform pore size distribution eq 30 in the range of Δ*r*_p_ = 0.5–15 nm and a mean pore diameter of *r*_p,0_ = 10 nm. As the liquid viscosity μ_l_ of an aqueous solution may change drastically during the drying due to the change of species concentration, two limit cases μ_l_ = 0.001 Pa·s and μ_l_ = 0.1 Pa·s were investigated. The results are shown in 2.

**Figure 2:**
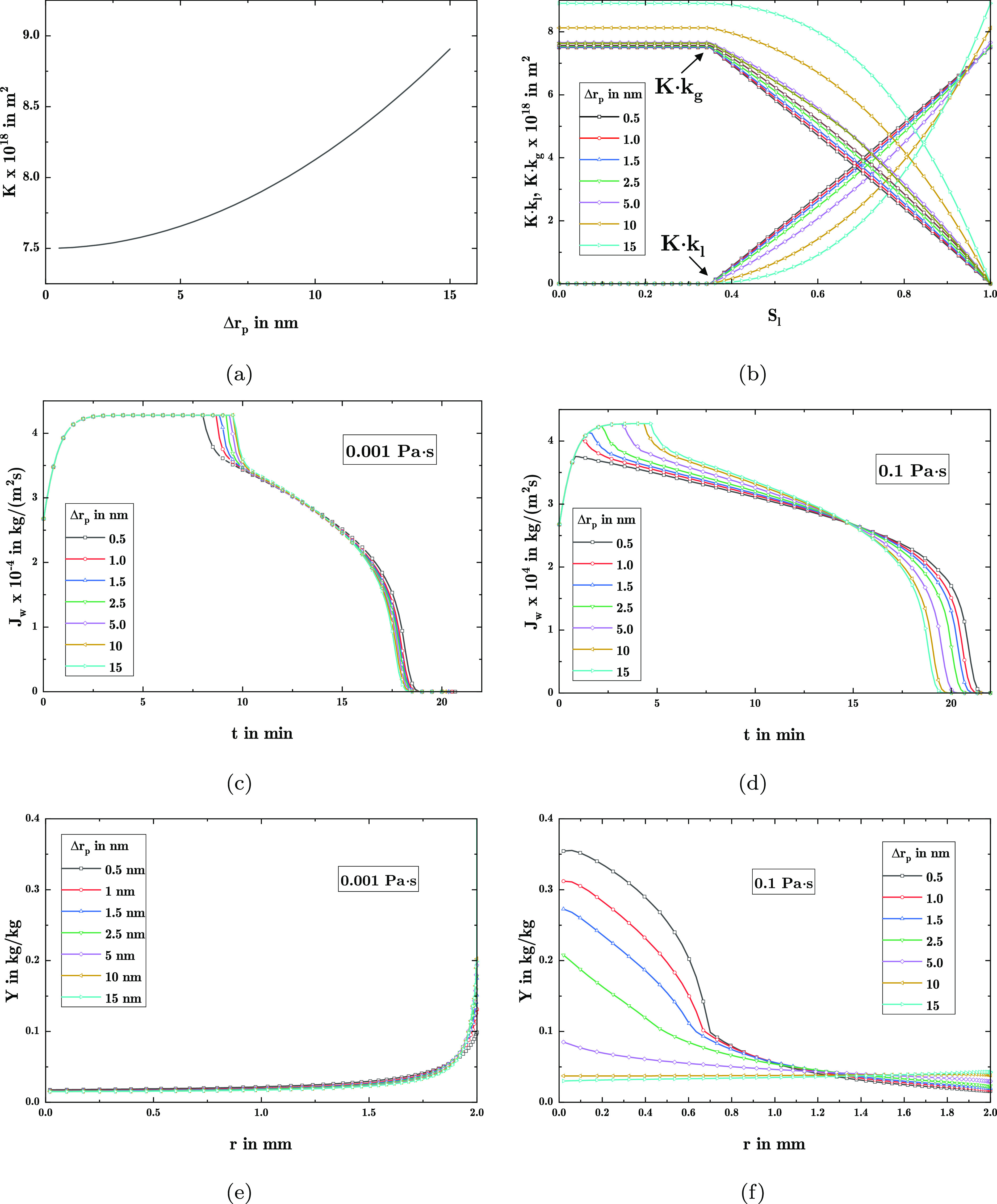
Drying with uniform distributions of varying widths at *T*_∞_ = 100 °C and liquid viscosity with μ_l_ = 0.001 Pa·s and μ_l_ = 0.1 Pa·s: (a) permeability over Δ*r*_p_, (b) partial permeability for varying Δ*r*_p_, (c,d) vapor flux across surface, and (e,f) precipitate load distribution.

There, the nonlinear correlation of the permeability *K* with the Δ*r*_p_ is visible in Figure 2a, leading to a significant increase of *K* with larger Δ*r*_p_. In Figure 2b, the phase permeability is displayed, represented by the product of the partial permeability *k*_i_ and total permeability *K* for the gas and liquid phase. There it is visible how an increasing Δ*r*_p_ leads to an increasingly nonlinear dependency of *K*·*k*_i_.

Furthermore, it can be observed in Figure 2c that the constant drying period shortens for a smaller Δ*r*_p_, leading to a generally longer drying time. In the case of μ_l_ = 0.1 Pa·s in Figure 2d, the constant drying period is shortened further compared to μ_l_ = 0.001 Pa·s, to such an extent that for Δ*r*_p_ ≤ 2.5 nm, no discernible constant drying rate period can be observed.

As for the distribution of the precipitate, for μ_l_ = 0.001 Pa·s in Figure 2e a strong tendency toward accumulation at the surface is apparent. The differences induced by varying Δ*r*_p_ between the precipitate profiles are comparatively small, showing only a small decrease in the tendency toward surface accumulation with broader pore size distributions. In contrast, for μ_l_ = 0.1 Pa·s in Figure 2f, a pronounced accumulation of the precipitate load toward the center of the sphere can be observed for increasingly narrow pore size distributions.

From these results, several insights can be gained: First, the presence of larger pores for larger Δ*r*_p_ leads to generally higher permeability and overall less resistance for gas transport, as *K*·*k*_g_ at larger Δ*r*_p_ always exceeds the values for smaller Δ*r*_p_. The liquid transport in contrast experiences higher resistance to convective flow for larger Δ*r*_p_ values for most of the liquid saturation range, except for *S*_l_ → 1. This is insofar sensible, as the liquid phase only at high liquid saturation is present in larger pores with less resistance and for lower values of *S*_l_ experiences the increased resistance by the smaller pores. As a result, a larger Δ*r*_p_ facilitates longer constant drying rate periods due to the initial presence of liquid in very large pores.

In contrast, once the falling drying rate period is entered, convective liquid transport toward the drying surface is higher for smaller Δ*r*_p_. Nevertheless, larger Δ*r*_p_ still facilitates faster drying for a significant part of the falling drying rate period, as the transport of vapor toward the drying surface is supported by larger pores. Subsequently, the pellets with larger pore-size distribution allow for faster drying, whereas a smaller distribution may increase the drying time considerable, as seen in 2d.

To arrive at a second insight, it is necessary to analyze the transport of aqueous precipitating species in eq 3. There, the only phenomena allowing redistribution are convective liquid flux toward the drying surface induced by capillary suction and a counter-diffusive flux toward the center of the pellet caused by the accumulation of species at the drying surface and the resulting gradient in the mass fraction. Thus, the induced change in precipitate distribution due to changes in μ_l_ are the result of a convectively dominated transport at μ_l_ = 0.001 Pa·s and higher influence of the diffusive fluxes at μ_l_ = 0.1 Pa·s. There, convective fluxes are suppressed due to the resistance induced by the higher viscosity, as also already visible in the reduction of the constant drying rate period. However, whereas the competition of convective and diffusive fluxes due to changes in liquid viscosity does explain the change in the global trend of the final precipitate distribution, it also needs to be taken into account that for smaller Δ*r*_p_ values, the overall drying time increases. This subsequently provides more time for diffusive flux to act and facilitates the trend toward less accumulation at the surface in 4c and increased accumulation of precipitate in the center of the pellet in 4d. Hence, it can be concluded that smaller Δ*r*_p_ reduces the liquid flux toward the surface and increases the drying time, which finally promotes a shift of the precipitate load distribution toward the center of the pellet.

### Distribution Type

**Figure 3:**
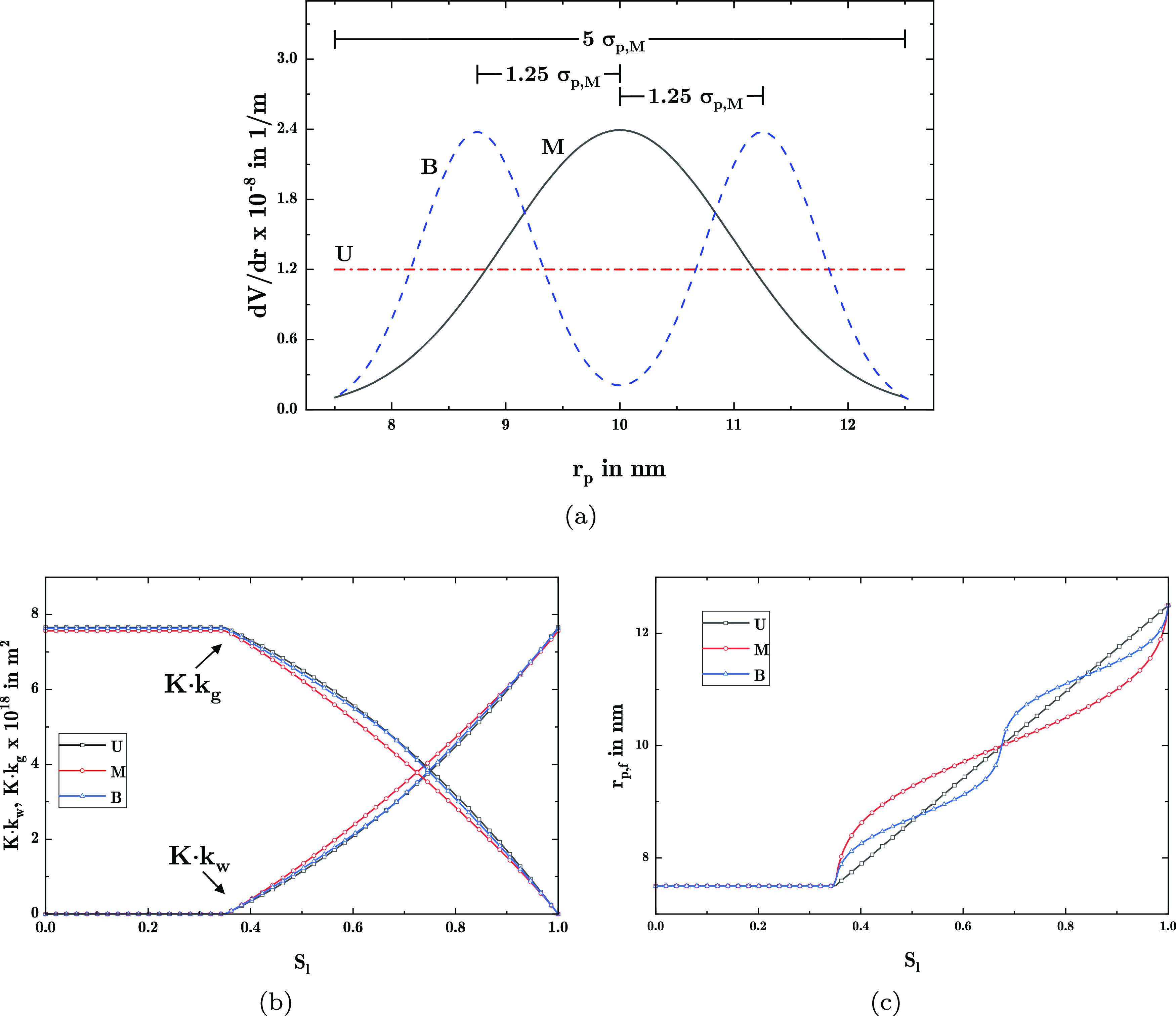
Comparison of uniform (U), monomodal (M), and bimodal (B) distribution within the evaluation range of *r*_p_ = 10 ± 2.5 nm: (a) normalized distribution, (b) phase permeabilities, and (c) representative radius with gas–liquid–solid contact line.

As a second step, comparable uniform, monomodal, and bimodal distributions were investigated. Comparability between the distribution types is given by evaluation within the same Δ*r*_p_ = 5 nm and the mean pore size diameter *r*_p_ = 10 nm, as shown in Figure 3a. The standard deviation of the monomodal distribution is set to σ_p,M_ = 1 nm, whereas for the bimodal distribution, the modes are chosen with a standard deviation of half of the monomodal one: σ_p,B_ = 0.5 nm. Additionally, the average pore radii of the bimodal distribution *r*_p,B_ are located at a distance of *r*_p,M_ ± 1.25 σ_p,M_.

Similar to the previous case, the drying was conducted with μ_l_ = 0.001 Pa·s and μ_l_ = 0.1 Pa·s. For the investigated distributions, the phase permeabilities in the form of *K*·*k*_i_ are shown in 3b, as well as the development of the largest filled pore radius *r*_p,f_ depending on the liquid saturation in 3c. There, the phase permeabilities of the bimodal distribution show almost no discernible difference in comparison to those derived from the uniform distribution. Only the monomodal distribution leads to a slight change in form and limit values of *K*·*k*_i_. In contrast, for *r*_p,f_ clear differences between the curves are introduced by the varying distribution types. Whereas the uniform distribution leads to a linear correlation between *r*_p,f_ and *S*_l_, the monomodal distribution invokes a dedicated profile, which is also seen for each individual model of the bimodal distribution.

**Figure 4:**
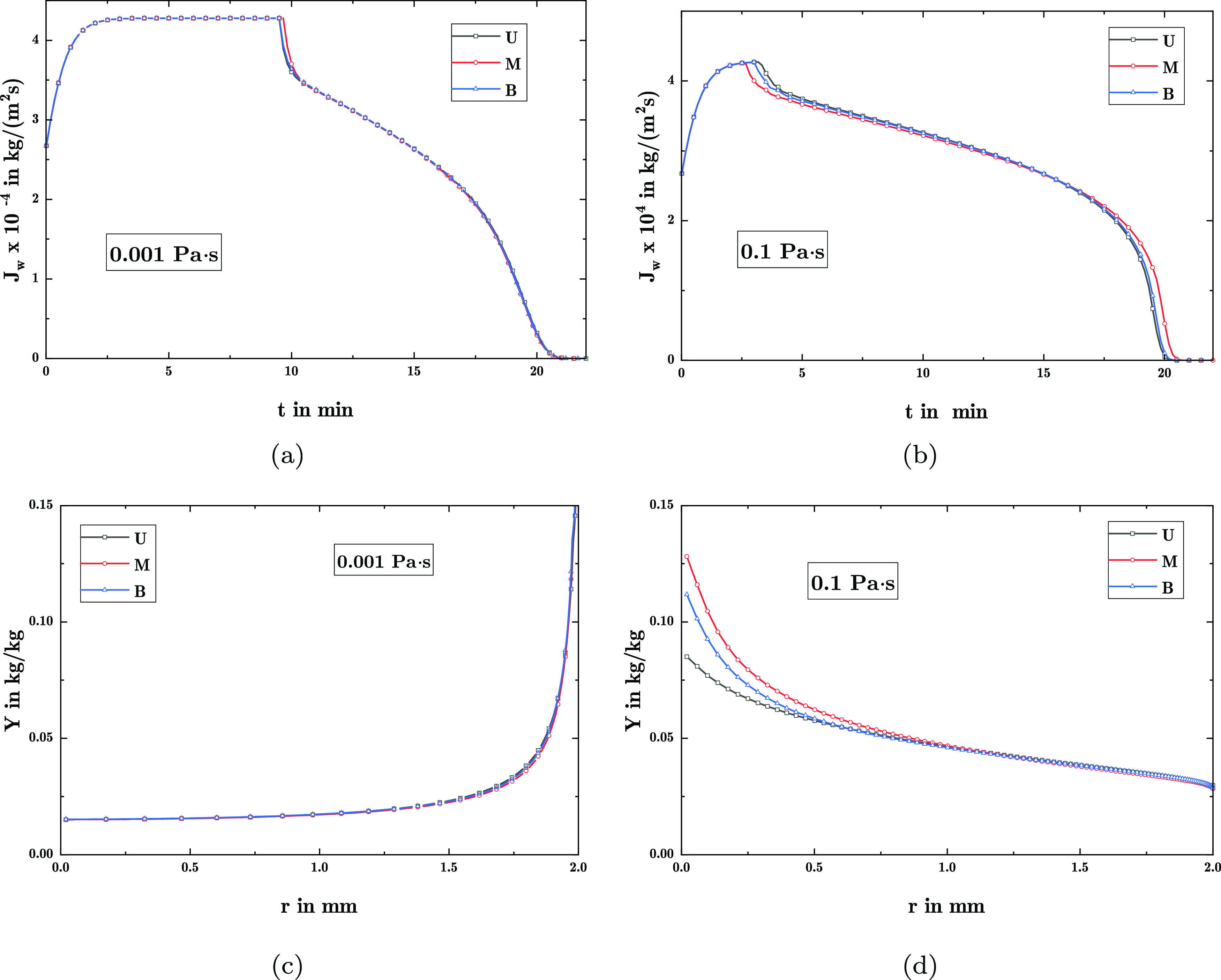
Drying with uniform (U), monomodal (M), and bimodal (B) distribution of same pore size spread Δ*r*_p_ at *T*_∞_ = 100 °C with the liquid viscosity values μ_l_ = 0.001 Pa·s and μ_l_ = 0.1 Pa·s: (a,b) vapor flux across surface and (c,d) precipitate load distribution.

The results for the effect of the distribution type on the drying and subsequent precipitate profiles are shown in Figure 4. Similar to the development of *K*·*k*_*i*_, the type of distribution in this setup has only a small effect on the development of the water vapor flux and the postdrying load distribution. Nevertheless, for μ_l_ = 0.1 Pa·s, an increased accumulation in the center of the pellet can be observed for the bimodal and monomodal distribution in contrast to the uniform distribution. Also, the development of the vapor flux at the surface slightly changes between the distribution types, leading to an earlier onset of the falling drying rate period for the monomodal distribution.

Following the earlier reasoning, the observed deviations are in accordance with the presence of larger pores. Within the bimodal and uniform distribution, more pore space is occupied by larger pores in contrast to the monomodal distribution. This in turn leads to the slightly higher permeability for bimodal and uniform distribution in 3b and, subsequently, to an extended constant drying rate period as well as faster drying. As the drying time increases for the monomodal distribution, the diffusive fluxes lead to a redistribution of the precipitation species toward the center of the pellet, observable for μ_l_ = 0.1 Pa·s in Figure 4d. Interestingly, the observed differences in profiles for *r*_p,f_, seem to have a negligible influence on the final redistribution of species, although they lead to a change in the capillary pressure according to eq 19. This may be attributed to the overall narrow width of the investigated distributions, which in this case leads to a comparatively narrow range of capillary pressure of 11.5 MPa ≤ *P*_c_ ≤ 19.2 MPa. Subsequently, the variations in *P*_c_ due to the different distribution types do not induce a distinguished change compared with the overall trends.

### Widely Spread Distributions

Here, the term “widely spread” distribution is applied to distributions, where *r*_p,min_ and *r*_p,max_ are separated by more than 1 order of magnitude. To investigate the influence of such distributions, a typical mesoporous support material is approximated by bimodal distributions according to eq 31 with *r*_p,1_ = 10 ± 1 nm and *r*_p,2_ = 1000 ± 100 nm. Such micrometer size pores could be introduced either on purpose during the synthesis of the support pellet or due to the formation of micro fractures during the impregnation step. Here, the evaluation range of the whole distribution is given by the limit values of evaluation of the separate modes, leading to 7.5 nm ≤ *r*_p_ ≤ 1250 nm. However, for such types of distributions, extended intervals of d*V*_p_/d*r*_p_ → 0 within *r*_p,min_ and *r*_p,max_ may be present, which then induce strong intermittent changes for *k*_l_, *k*_g_, and *r*_p,f_ in eqs 22, 23, and 25. This significantly increases the stiffness of the formulated problem, which may not be desirable. To improve the convergence rate of the simulations, a small fraction *f*_U_ of the pore volume is converted into a uniform distribution spanning the whole interval of interest. Within this investigation, pore size distributions are considered to be formed by a combination of eqs 30 and 31
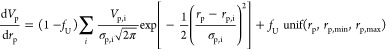
32

**Figure 5:**
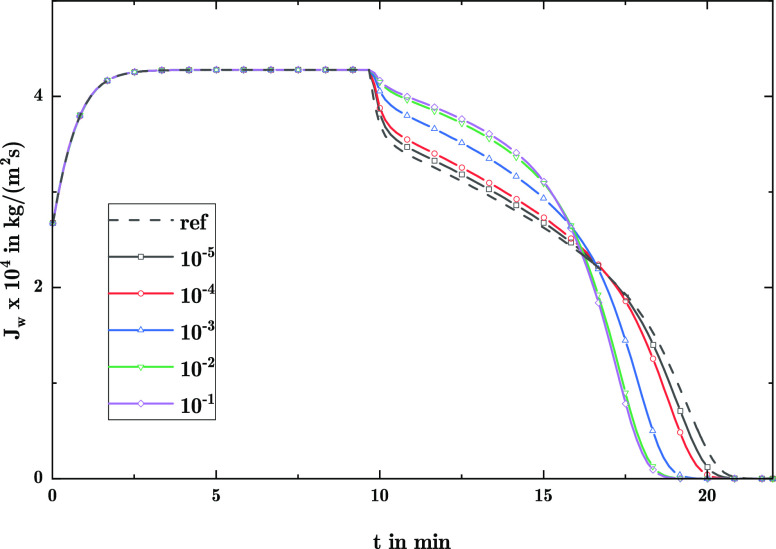
Water vapor flux *J*_w_ over time with varying *f*_U_ compared against a purely monomodal distribution (ref).

To determine a fitting value for *f*_U_, a pseudo monomodal distribution with *V*_p,2_ = 0 is subjected to drying. The temporal development of the vapor flux at the drying surface is compared to the above investigated corresponding monomodal distribution, as shown in Figure 5. There it is clearly visible how small values of *f*_U_ can have a rather significant effect on the falling drying rate period. For this investigation, a deviation of *f*_U_ = 10^–5^ was considered acceptable.

The influence of widely spread distributions on the drying behavior and resulting precipitate redistribution is investigated by adapting the fractional pore volumes of the two modes. As this research focuses on predominantly mesoporous media (*r*_p_ < 50 nm) for supported catalysts, the void volume with larger characteristic pore radii is limited to *V*_p,2_ ≤ 0.5 *V*_p,1_.

**Figure 6:**
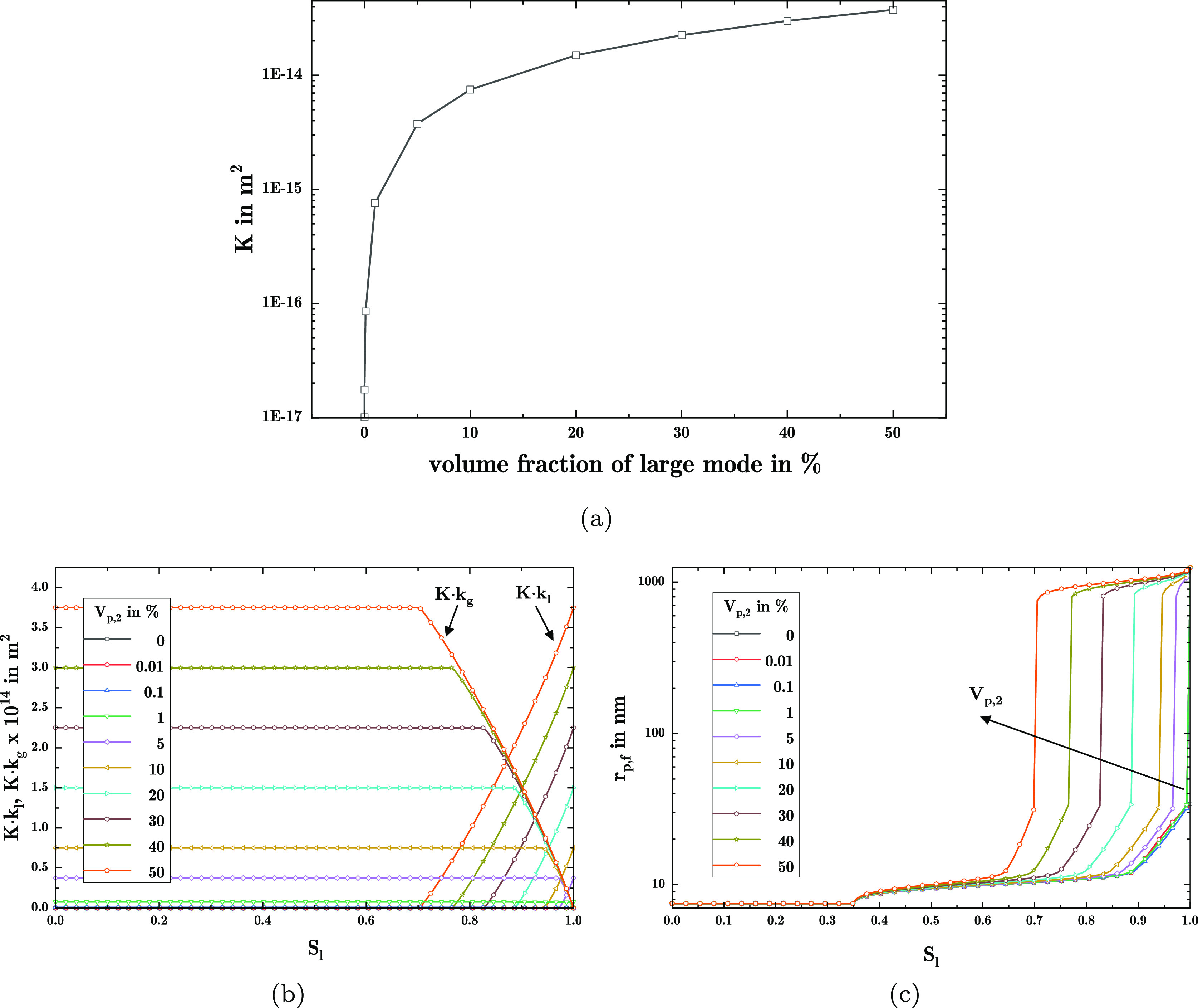
Derived properties of the widely spread distributions: (a) total permeability, (b) phase permeability, and (c) largest filled radius.

The total permeability *K*, phase permeability *k*_i_·*K*, as well as the largest filled pore radius *r*_p,f_ are shown in Figure 6.

Figure 6a shows how already small volume fractions with large pore sizes lead to a strong change in permeability, effectively increasing it by almost 3 orders of magnitude within the range 0 < *V*_p,2_ < 0.1. Furthermore, Figure 6b indicates the distribution of the permeability for the gas and liquid phase. There it is visible that for a higher *V*_p,2_, the interval of high permeability for the liquid phase increases. In contrast, with a lower *V*_p,2_, the liquid phase experiences only high phase permeabilities at higher liquid saturation levels. Similarly, the development of *r*_p,f_ with liquid saturation also changes with adapting *V*_p,2_. There, a large jump is observable for saturation levels close to the change between modes in the pore size distribution, moving toward lower saturation levels with increasing *V*_p,2_. As a second observation, the change of *r*_p,f_ further away from the jump stays qualitatively the same for all distributions.

The observed behavior leads to several conclusions: especially gas flow experiences significantly higher permeability for already small amounts of large pores. In contrast, for liquid flow, the permeability is dictated by the smaller pores for low liquid saturation levels. Only for comparatively large fractions of pore space with large pore radii and then at high liquid saturation levels can liquid flow benefit from higher permeability. Another insight can be derived from 6c for the development of the liquid flow due to induced gradients in capillary pressure. Especially at the jump region between the two modes, large gradients of capillary pressure are induced by the local change of *r*_p,f_, potentially leading to high local capillary suction. However, outside of this region, gradients in *r*_p,f_ are comparatively low, invoking lower gradients in the capillary pressure and finally lower values for the associated liquid fluxes.

**Figure 7:**
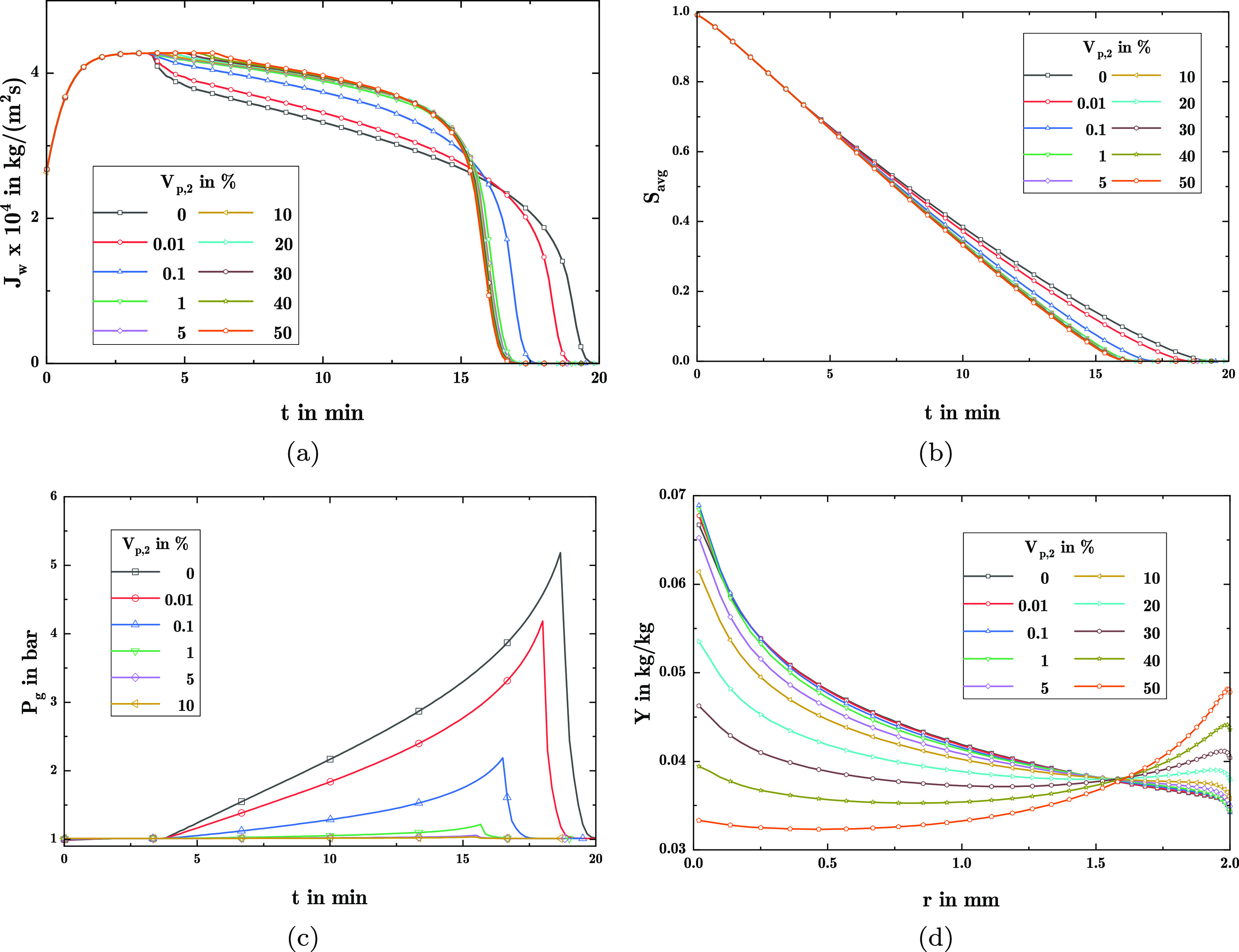
Results for drying with widely spread pore size distributions with varying *V*_p,2_: (a) water vapor flux at the surface, (b) average saturation, (c) gas pressure at center of the sphere, and (d) precipitate load distribution.

The impact on the drying process with those distributions is shown in Figure 7.

There, Figure 7a,b displays a visible change in the form of the vapor flux, and the constant drying rate period is extended by the presence of large pores. Similarly, a significantly shorter drying time already for relatively low volume of pores with large radii is induced. Furthermore, the maximum reached gas pressure at the center of the porous pellet during the drying is substantially decreased by the presence of larger pores. Finally, larger pores also lead to a substantial change in the postdrying load distribution of the precipitated species in Figure 7d. There, a change from accentuated accumulation in the center of the sphere toward major precipitation at the surface is induced, albeit only for comparatively large volume fractions of larger pores.

From these observations, it can be concluded that the influence of pore volume fractions with larger pore radii is 2-fold: first, the larger pores allow for higher vapor fluxes toward the surface and less gas pressure drop due to the lower drag by the larger pores. Subsequently, the thus induced higher vapor fluxes then lead to faster drying of the porous pellet. However, the decrease in drying time is mainly apparent for 0 < *V*_p,2_ < 0.01, where no pronounced difference in precipitate load is observable. In contrast, for larger partial volumes of larger pores, drying conducts at comparable rates whereas species is redistributed significantly. This is caused by an increased occupancy of large pores with liquid, which thus experiences a significantly higher permeability, as shown in Figure 6b. As a result, the convective fluxes by capillary suction toward the surface can be continued longer, thus leading to the increased accumulation at the surface and the extended constant drying rate period.

Second, even low amounts of larger pores act as the main path for the gas flow and subsequently prevent high gas pressure build ups at the center of the particle. This is insofar of interest, as the induced stress on the material by the gas pressure may induce fractures or even disintegration of the porous pellet during the drying.

## Conclusions

As a part of this work, an averaged model describing drying in porous media was extended with mass transfer and precipitation. Additionally, an analytical permeability model based on the pore size distribution was employed to derive the hydrodynamic properties of the associated model geometry. Subsequently, this model has been used to study the influence of varying types of pore size distributions, as well as the impact of large differences in pore sizes on the drying process and the redistribution and consecutive precipitation of an aqueous species in mesoporous media, i.e., supported catalyst supports.

From the presented results, a variety of insights can be derived. First, for comparatively narrow pore size distributions on the mesoscale, the exact form of the pore space only shows a significant impact for rather viscous liquids. There, a wider pore size distribution leads to faster drying, whereas for narrower ones, a higher accumulation of species toward the center is induced. However, for water and liquids with comparable viscosity, the form and width of pore size distribution only have a rather small effect on the final distribution of the precipitated species.

This is directly correlated with the second insight that the presence of larger pores facilitates increased gas flow, allowing for faster removal of vapor from the porous media. In contrast, the presence of smaller pores leads to a higher resistance against liquid flow, which is partially alleviated by an increase in capillary suction and the presence of a gas pressure gradient. Within the limitations of the presented model formulation, it can be concluded that the presence of even a small amount of large pores acts as a major pathway for gas flow, leading to a significant decrease of pressure build up within the porous media during the drying process and shortening of the required drying time, while having an almost negligible impact on the final distribution of the precipitated species. However, this is true only if the volume of large pores is not occupied by liquid throughout the majority of the drying. Once a significant amount of liquid is present within larger pores, redistribution of the species is influenced noticeably, inducing an increased transport toward the drying surface.

Following these conclusions, a third insight can be derived. As initially mentioned, popular permeability models often rely on a certain form of the pore size distribution. However, it can be concluded from the results that especially for wide distributions, the detailed knowledge of the pore space morphology can be crucial to predict and control the drying behavior as well as the final distribution of the precipitated species within the porous material.

At this point, it is necessary to emphasize that species transport and precipitation in commonly used porous catalyst support media, i.e., zeolites or alumina, is subject to a variety of further influences, such as adsorption on amphoteric surfaces, non-Newtonian fluids, transport in confined pore spaces, and electrostatic interactions between multiple aqueous species to name a few.

To further understand the redistribution and deposition of aqueous species during the drying of supported catalysts, it would be highly interesting to understand the impact of dynamic viscosity changes in the dependency of concentration of species and their interplay with percolation phenomena as well as incorporation of pore scale redistribution events. Additionally, investigating the relevance of descriptions for gas flow in the Knudsen regime would be pertinent, especially its impact on the internal pressure build up, the induced liquid flux, and subsequent redistribution of the aqueous species.^29^ Finally, another intriguingly complex interplay of transport phenomena is given by the change in local pore morphology due to the accumulation of the precipitate, as it commonly can be observed on the pellet scale with efflorescence.^30^

## Supporting Information Available

Reference to a repository with raw data and scripts, numerical details and validation of the discussed model, and complete set of constitutive equations utilized within the discussed model (PDF)
Reference files (ZIP)

## Nomenclature

α: external heat transfer coefficient in W/m^2^
β: external mass transfer coefficient in m/s
ε: volume fraction
ζ: spatial variable.
θ: contact angle
λ: thermal conductivity in W/(m K)
μ: dynamic viscosity in Pa·s
ρ: density in kg/m^3^
σ: surface tension in N/m
σp: standard deviation of pore size distribution in m
ϕ: arbitrary variable
ω: mass fraction
*v*: velocity in m/s
cp: isobaric heat capacity in J/(kg K)
*D*: effective diffusion coefficient in m^2^/s
*E*: conservation error
*h*: specific enthalpy in J/kg
*J*: flux in kg/(m^2^s)
*K*: permeability in m^2^
*k*: partial permeability
*k*_prec_: precipitation reaction rate constant in s^–1^
*M*: molar mass in mol/kg
*P*: pressure in Pa
*P*_∞_: ambient pressure in Pa
*R*: radius sphere in m
*r*: distance to sphere center in m
*S*: saturation
*T*: temperature in K
*t*: time in s
*V*: volume in m^3^
*V*_p_: normalized pore volume
*Y*: load of precipitate in kg/kg
