# Supplementary material for: Modeling the Drying Process of Porous Catalysts: Impact of the Pore Size Distribution
Source: Ind Eng Chem Res. 2023 Nov 14;62(46):20006–16. doi: 10.1021/acs.iecr.3c03057 (PMC10682989; doi:10.1021/acs.iecr.3c03057)
Supplement: Supplementary file 1 — ie3c03057_si_001.pdf [file ie3c03057_si_001.pdf]

Supporting Information

Modeling the drying process of porous catalysts - impact of the pore size distribution

David R. Rieder, Elias A.J.F. Peters*, Johannes A.M. Kuipers

Multiphase Reactors Group, Department of Chemical Engineering and Chemistry, Eindhoven University of Technology, P.O. Box 513, 5600 MB Eindhoven, The Netherlands

*Corresponding author (e.a.j.f.peters@tue.nl)

Availablility of scripts and files

The associated matlab scripts, input files, generated data and evaluation procedures are available free of charge on https://data.4tu.nl with DOI: 10.4121/b62c98f8-be8c-4a4a-9d7f-b25915d47745

Numerical Details & Validation

Solving scheme

The governing equations form a system on non-linear partial differential equations and cannot be solved analytically. Therefore, a numerical approach was chosen where the equations are spatially discretized with the finite volume method and Euler-backward for temporal resolution. As a means to determine the solution for each timestep, Newton-Raphson iterations were employed within a Mathworks® MATLAB implementation. The associated Jacobian is determined via numerical differentiation of the residuals with a step size of 10^{-10} relative to the field value. For the convective fluxes of the species transport, a MINMOD TVD scheme was applied.

Additionally, the model has been found to be sensitive to strong gradients in the liquid saturation S_l, which predominantly appear during the initial time steps near the drying surface. The thus induces sharp changes in local parameters may induce numerical oscillations, if the gradients are not resolved sufficiently. To suppress those oscillations, the numerical grid is

refined towards the surface with along the dimensionless spatial variable ζ:

$$\zeta = r/R \tag{S1}$$

$$\Delta\zeta(n) = \frac{(1+\Delta\zeta_{min}) - \zeta(n)}{\sum_n^{n_{tot}}(1+\Delta\zeta_{min}) - \zeta(n)} \tag{S2}$$

with the cell index n, the total number of cells n_{tot} and minimal relative grid spacing $\Delta\zeta_{min}$ at $\zeta \longrightarrow 1$. For this investigation, a grid resolution of $n_{tot} = 100$ cells and $\Delta\zeta_{min} = 0.01$ were considered sufficient.

Numerical treatment of pore size distributions

As a means to investigate any arbitrary pore size distribution, equation equation (S3) - (S7) are evaluated numerically.

$$K = \frac{1}{8}\int_{r_{p,min}}^{r_{p,max}} r^2 \frac{dV_p}{dr_p}\mathrm{d}r_p \tag{S3}$$

$$k_l = \frac{1}{8K}\int_{r_{p,min}}^{r_{p,f}} r_p^2 \frac{dV_p}{dr_p}\mathrm{d}r_p \tag{S4}$$

$$k_g = \frac{1}{8K}\int_{r_{p,f}}^{r_{p,max}} r_p^2 \frac{dV_p}{dr_p}\mathrm{d}r_p \tag{S5}$$

$$k_l + k_g = 1 \tag{S6}$$

$$S_{l,f} = \frac{S_l - S_{l,crit}}{1 - S_{l,crit}} = \frac{1}{1-\epsilon_s}\int_{r_{p,min}}^{r_{p,f}} \frac{dV_p}{dr_p}\mathrm{d}r_p \tag{S7}$$

However, constant integration of those functions throughout the solution requires significant amounts of computational time. As a means to approximate k_l, k_g and $r_{p,f}$, while keeping the introduced error low, satisfy smoothness and significantly decrease the required computation time, modified Akima piecewise cubic Hermite interpolation is applied [1], as provided by the *makima* function of MATLAB. Additionally, under- and overshoots of k_l, k_g and r_f

have to be avoided at $S \approx S_{crit}$. This is guaranteed by extending the initially integrated data set to $S < S_{crit}$ by two points into $S < S_{crit}$ with the respective limit values $k_l = 0$, $k_g = 1$ and $r_{p,f} = r_{p,min}$

Numerical treatment of permeability model

To verify its implementation, the results of the permeability model are compared with the analytical solution for a homogeneous pore-size distribution and with published data for mono- and bimodal distributions by Vu & Tsotsas [2]. For the uniform distribution, following expression was employed:

$$\frac{dV_p}{dr_p} = \frac{0.6}{r_{p,max} - r_{p,min}} \tag{S8}$$

whereas the mono- and bimodal distributions follow:

$$\frac{dV_p}{dr_p} = \sum_i \frac{C_i}{\sigma_{p,i}\sqrt{2\pi}} \exp\left(-\frac{1}{2}\left(\frac{r_p - r_{p,i}}{\sigma_{p,i}}\right)^2\right) \tag{S9}$$

with the number of modes i, their respective volume C_i, mean pore diameter $r_{p,i}$ and standard deviation $\sigma_{p,i}$. The respective details are shown in table S1.

case	C_0	$r_{p,0} \pm \sigma_{p,0}$ in nm	C_1	$r_{p,1} \pm \sigma_{p,1}$ in nm
V1	1	100 ± 5	-	
V2	1	1000 ± 100	-	
V3	0.5	100 ± 10	0.5	200 ± 20
V4	0.5	100 ± 10	0.5	2000 ± 200

Table S1 pore size distribution data for the verification cases [2].

The numerically derived K, k_w and k_g for equation (S8) in the range of $0.1 \text{ m} \leq r_p \leq 1 \text{ m}$ were compared with their analytically derived solutions:

$$K = 0.0277 \text{ m}^2 \tag{S10}$$

$$k_l(S_{l,f}) = (S_{l,f}(r_{p,max} - r_{p,min}))^3 \frac{1}{\text{m}^3} \tag{S11}$$

$$k_g(S_{l,f}) = 1 - k_w(S_{l,f}) \tag{S12}$$

$$r_{p,f}(S_{l,f}) = r_{p,min} + S_{l,f}(r_{p,max} - r_{p,min}) \tag{S13}$$

The numerically determined permeability K shows a relative deviation of $1.52 \cdot 10^{-4}$. For k_w, k_g and r_f the results are given in figure S1c and S1b. Their respective L_2-errors both lie well below $1 \cdot 10^{-4}$. As for the monomodal and bimodal cases in table S1, the results are shown in figure S1c and S1d and show an overall maximum L_2-error below $5 \cdot 10^{-3}$. With those results, implementation is considered to be sufficiently accurate and verified.

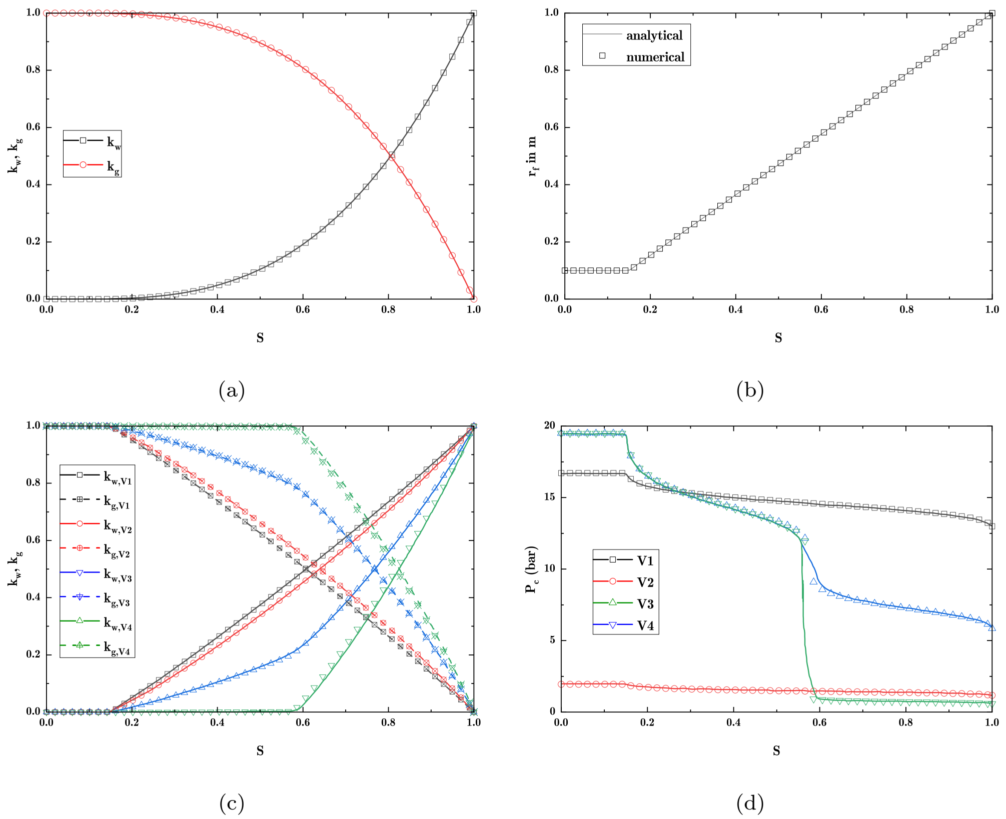

Figure S1 Results for the verification of the permeability model: (a) partial permeabilities and (b) largest filled radius $r_{p,f}$ of the uniform distribution, as well as (c) partial permeabilities and (d) capillary pressure by [2]; case V1 - V4 are given in figure S1.

Drying of light concrete

Verification of the drying model has been conducted against the results provided in [3] and [2]. There, the drying of a spherical pellet of light concrete has been simulated. In the first case (I), the drying is conducted at $T_\infty = 20$ °C with the relative humidity of 50 % in the drying air and highlights the temperature and water flux, as seen in figure S2. In contrast, the second case (II) conducts the drying at $T_\infty = 80$ °C in dry air. The transient profiles of

the moisture content and pressure, shown in figure S3.

In both cases, a very good qualitative and quantitative agreement with the reference data is reached. Only in figure S3b a slight deviation from the reference data near the end of the drying can be observed, leading to a slightly faster occurrence of the drying. However, the model is quite sensitive towards the exact form of the underlying correlations, discretization and averaging approaches, as well as the uncertainty of the exact determination procedure of the reference data, the implementation is considered sufficiently verified.

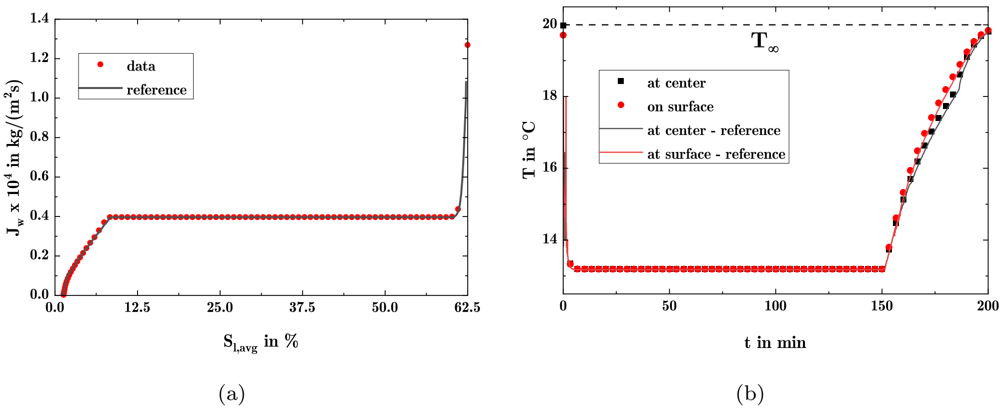

Figure S2 Results for verification case I [2]: vapor flux and b) temperature at the center and surface of the particle.

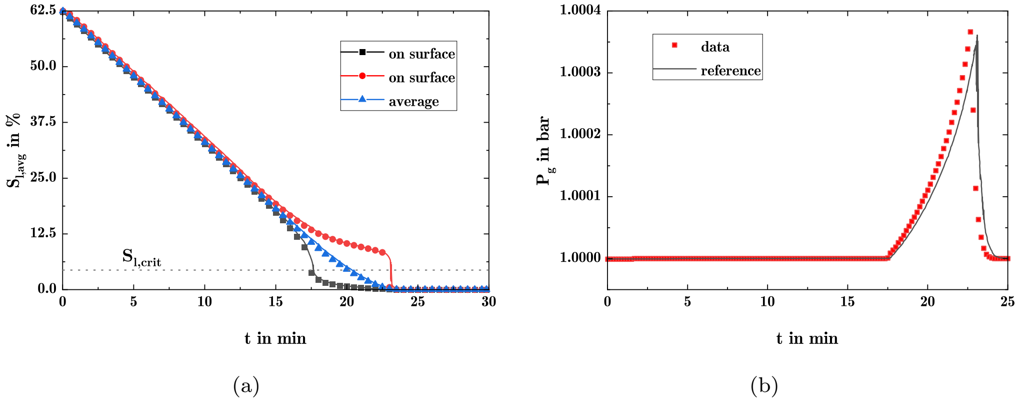

Figure S3 Results for verification case II: a) saturation content and b) gas pressure at the center of the particle. Symbols denote the generated data, line the reference taken from [3].

Properties and correlations

Parameters used for the investigation

An overview over the transport coefficients and other simulation parameters is provided in table S2.

Symbol	value	meaning
$\alpha =$	14.25 W/m^2	external heat transfer coefficient
$\beta =$	0.015 mol/(m^{2}s)	effective mass transfer coefficient
$\epsilon_s =$	0.4	volume fraction of solid
$S_{crit} =$	0.35	critical moisture content
$S_{init} =$	0.99	initial saturation
$\rho_l =$	1000 kg/m^3	liquid density
$\rho_s =$	2500 kg/m^3	solid density
$\mu_{l,0} =$	0.001 Pa $\cdot$ s	viscosity of pure water
$\mu_g =$	$14.626 \cdot 10^{-6}$ Pa $\cdot$ s	gas viscosity
$M_a =$	29 g/mol	molar weight of air
$M_v =$	18 g/mol	molar weight of water
$M_n =$	130 g/mol	molar weight of transported species
$h_l =$	4190 J/(kgK)$(T - T_{ref})$	enthalpy of the liquid
$h_s =$	960 J/(kgK)$(T - T_{ref})$	enthalpy of the solid
$h_v =$	1874 J/(kgK)$(T - T_{ref})$	enthalpy of the vapor
$h_a =$	1006 J/(kgK)$(T - T_{ref})$	enthalpy of the air
$k_{prec} =$	10^4 1/s	precipitation reaction rate constant
$\Delta h_v =$	$-2.5 \cdot 10^6$J/kg	evaporation enthalpy
$c_{sat} =$	5 mol/l	saturation concentration
$x_{sat} =$	0.65	weight fraction of species at saturation
$D_{wn} =$	$1.67 \cdot 10^{-9}$ m^2/s	effective diffusion coefficient of species
$T_{ref} =$	273.15 K	reference temperature
$P_{ref} =$	101300 Pa	reference pressure

Table S2 Parameters used during the investigation.

The effective diffusion coefficient of vapor in the gas phase is determined by linear scaling with the area available for the flux:

$$D_g = \epsilon_g D_{g,bin} \tag{S14}$$

with the binary diffusion coefficient $D_{g,bin}$ of vapor in air. Note, that tortuosity is neglected, as it would merely act as an additional scaling factor without additional merit to this work. Similarly, the effective diffusion coefficient of the liquid $D_{l,wp}$ is computed by:

$$D_{l,wp} = S_{l,f}(1 - \epsilon_s) D_{l,wp,bin} \tag{S15}$$

with the constant binary diffusion coefficient of the dissolved species in water $D_{l,wp,bin}$. The effective thermal conductivity is computed as arithmetic average quantity and by neglecting conduction in the gas phase:

$$\lambda = \epsilon_s \lambda_s + \epsilon_l \lambda_l \tag{S16}$$

The enthalpy of all the solid, water, precipitate and air is computed by:

$$h_i = c_{p,i}(T - T_{ref}) \tag{S17}$$

where T_{ref} denotes a reference temperature and $c_{p,i}$ the relevant heat capacity. For the vapor phase, the evaporation enthalpy Δh_v is additionally taken into account:

$$h_{g,w} = c_{p,v}(T - T_{ref}) + \Delta h_v \tag{S18}$$

The vapor pressure is computed according to the Antoine-equation:

$$P_v = 133.32\ \exp(18.584 - 3984.2/(T - 39.724))\ \mathrm{Pa} \tag{S19}$$

For the binary diffusion coefficient of vapor in air, following expression holds:

$$D_{va} = 2.26 \cdot 10^{-5}\ (T/T_{ref})^{1.81} P_{ref}/P_g\ \mathrm{m^2/s} \tag{S20}$$

Supplements verification cases

If not explicitly mentioned, the same parameter as discussed above apply. In both verification cases, the drying of light concrete is investigated. An overview of the relevant parameters is provided in table S3.

Symbol	value	meaning
$\epsilon_s =$	0.2	solid volume fraction
$K =$	$2 \cdot 10^{-13}\text{m}^2$	permeability
$k_l =$	S_{fw}^3	liquid side partial permeability
$k_g =$	$1 + (2S_{fw} - 3)S_{fw}^2$	gas side partial permeability
$R =$	2.5 mm	radius of the sphere
$S_{init} =$	0.625	initial saturation
$\lambda_l =$	$0.142 + 0.736\epsilon_l/(1 - \epsilon_s)$	thermal conductivity of the liquid
$c_{init} =$	0 mol/l	initial concentration of aqueous species

Table S3 Parameters used during the verification.

The liquid viscosity μ_l was calculated as follows:

$$\mu_l = -1.27 \cdot 10^{-9}(T - 273.15\text{ K})^3 + 3.42 \cdot 10^{-7}(T - 273.15\text{ K})^2 \quad \text{(S21)}$$

$$-3.43 \cdot 10^{-5}(T - 273.15\text{ K}) + 1.56 \cdot 10^{-3}\text{ Pa} \cdot \text{s} \quad \text{(S22)}$$

The capillary pressure was computed in dependence of the local saturation:

$$P_c = 40\sigma \exp\left(8.4057x10^{-0.5562(S-0.0438)}\right)\text{ Pa} \quad \text{(S23)}$$

and the surface tension in dependence of the local temperature:

$$\sigma = -1.3 \cdot 10^{-7}(T - 273.15\text{ K}) \quad \text{(S24)}$$

$$-1.85 \cdot 10^{-4}(T - 273.15\text{ K}) + 0.07606\text{ N/m} \quad \text{(S25)}$$

References

[1] Akima, H. A New Method of Interpolation and Smooth Curve Fitting Based on Local Procedures. Journal of the ACM **1970**, 17, 589–602.

[2] Vu, H. T.; Tsotsas, E. A Framework and Numerical Solution of the Drying Process in Porous Media by Using a Continuous Model. International Journal of Chemical Engineering **2019**, 2019.

[3] Vu, H. T.; Tsotsas, E. Mass and Heat Transport Models for Analysis of the Drying Process in Porous Media: A Review and Numerical Implementation. International Journal of Chemical Engineering **2018**, 2018, 1–13.

This information is available free of charge via the Internet at http://pubs.acs.org/
